# 

**Published:** 1837-04-01

**Authors:** 


### CONTENTS

OF THE

### MEDICO-CHIRURGICAL REVIEW,

### No. LII. APRIL 1, 1837,

### [BEING No. XII. OF A DECENNIAL SERIES.]

### REVIEWS.

I.

PATHOLOGICAL ANATOMY.

I. Outlines of Human Pathology. By HERBERT MAYO, Esq. Parts I. and II. ..
II. Lectures on the Nervous System and its Diseases. By MARSHALL HALL, M.D. &c.
III. The Human Brain, its Configuration, Structure, Development, and Physiology; illustrated by References to the Nervous System in the Lower Orders of Animals. By SAMUEL SOLLY, Esq. ..
IV. Lectures on the Morbid Anatomy of the Serous and Mucous Membranes. By THOMAS HODGKIN, M.D. ..
V. Outlines of Physiology. By HERBERT MAYO, Esq. ..
VI. Pathological and Surgical Observations relating to Injuries of the Spinal Cord. By Sir B. BRODIE ..
VII. Illustrations of the Elementary Forms of Disease. By R. CARSWELL, M.D. .. } 297

1. Pathology of the Spinal Cord .. 298

2. Injuries of the Spinal Cord .. 306

   A. Wounds of the Spine .. 306

   B. Concussion of the Spinal Cord .. 308

   C. Symptoms dependent on Injuries of the Cord .. 310

   D. Treatment of Injuries of the Spine .. 322

3. Parasitical Animals .. 325

   A. Of Vesicular Worms or Hydatids .. 326

   B. Of Flat Worms .. 329

   C. Of Cylindrical Worms .. 330

4. Analogous Tissues .. 333

   A. Origin of Analogous Tissues .. 333

   B. Erectile Tissue .. 337

   C. Cellular Tissue .. 338

   D. Serous Tissue .. 338

   E. Mucous Tissue .. 339

   F. Cutaneous Tissue .. 340

   G. Fibrous, Fibro-Cartilaginous, and Osseous Tissues .. 340

   H. Analogous Transformations .. 343

II.

VITAL STATISTICS.

I. A Statistical Account of the British Empire. By J. R. M'CULLOCH ..

II. Medical Statistics, or the Causes and Effects of the Extension of Human Life. By J. F. PALMER .. } 345

III.

A Practical Treatise on the Management and Diseases of Children. By R. T. EVANSON, M.D. and HENRY MAXWELL, M.D. .. 362
1. Infantile Therapeutics .. 365
2. Diseases of the Digestive Organs .. 366

IV.

A Clinical Treatise on the Endemic Fevers of the West Indies. By W. J. Evans, Esq. 368

V.

Medical Education.

Medical Study; an Introductory Address delivered at the British School, at the Opening of the Winter Session of 1836. By J. A. Symonds .. .. .. .. .. .. .. 375

1. Necessity of General Knowledge, and of a Scientific Character .. .. .. 376
2. Mode of Professional Study .. .. .. .. .. .. .. .. .. .. .. .. 381
3. The Spirit of Professional Study .. .. . .. .. .. .. .. .. .. 385

VI.

Essai sur la Philosophie Medicale. M. Bouillaud on Medical Philosophy .. .. .. 386

VII.

Lectures illustrative of certain Local Nervous Affections. By Sir B. C. Brodie .. .. 408

1. Theory of Local Nervous Affections—Various circumstances under which they exist—Principles on which the Treatment of them should be conducted .. 405
2. Various Forms of Local Hysterical Affections .. .. .. .. .. .. .. .. 418

VIII.

Public Health.

Hygiene Publique. Par A. J. B. Parent Duchatelet .. .. .. .. .. .. .. .. 423

### PERISCOPE.

### Bibliographical Notices.

1. A Practical Treatise on the Principal Diseases of the Lungs. By G. H. Weatherhead, M.D. .. .. .. .. .. .. .. .. .. .. .. .. .. .. .. .. .. 441
2. Practical Observations on Nervous and Sympathetic Palpitation of the Heart. By J. C. Williams, M.D. .. .. .. .. .. .. .. .. .. .. .. .. .. .. 442
3. The Hunterian Oration, delivered 14th Feb. 1837. By Sir B. C. Brodie, Bart. .. 445
4. Compendium of Lithotripsy. By Henry Belinaye, Esq. .. .. .. .. .. .. .. 448
5. Recent Publications on Anatomy .. .. .. .. .. .. .. .. .. .. .. .. .. 451
6. New Pharmaceutical Works.. .. .. .. .. .. .. .. .. .. .. .. .. .. .. 452
7. Principal Original Papers published in the Weekly and Monthly British Journals, between 1st Jan. and 20th March, 1837. .. .. .. .. .. .. .. .. .. .. 452

### Spirit of the English Periodicals, and Notices of English Medical Literature.

1. Recent Researches on Anatomy.
   1. Observations on the Thyroid Gland. By Mr. T. W. King.. .. .. .. .. .. 457
      A. The Thyroid Gland made up of Lobes and Cells containing a Fluid .. .. 457
      B. Analysis of the Fluid—Vessels and Nerves of the Gland .. .. .. .. .. 458
      C. Relative Position of the Gland—its frequent Compression .. .. .. .. 459
      D. Uses of the Gland .. .. .. .. .. .. .. .. .. .. .. .. .. .. .. .. 460
   2. Notes on the Structure of the Thyroid Gland. By Sir Astley Cooper, Bart. .. 460
      A. Thyroid Gland of the Cat .. .. .. .. .. .. . .. .. .. .. .. 460
      B. Thyroid Gland of the Dog .. .. .. .. .. .. .. .. .. .. .. .. .. 461
      C. Thyroid Glands of the Sheep.. .. .. .. .. .. .. .. .. .. .. .. .. 461
      D. Thyroid Glands of the Horse.. .. .. .. .. .. .. .. .. .. .. .. .. 461
      E. Thyroid Gland of the Pig .. .. .. .. .. .. .. .. .. .. .. .. .. 461
      F. Morbid Anatomy of the Thyroid Gland .. .. .. .. .. .. .. .. .. 462
   3. The History of an Unusually Formed Placenta, and Imperfect Fœtus, and of similar examples of Monstrous Productions. By Dr. Hodgkin, with an Account of the Structure of the Placenta and Fœtus. By Sir A. Cooper.. .. 462
      A. Structure of the Imperfect Fœtus.. .. .. .. .. .. .. .. .. .. .. 464
   4. On the Brain of the Negro compared with that of the European and of the Ourang Outang. By F. Tiedemann, M.D... .. .. .. .. .. .. .. .. .. 467
   5. Recent Investigations into the Composition of the Brain and Spinal Cord .. 468
   6. Anatomical Remarks on Hunchbacks .. .. .. .. .. .. .. .. .. .. .. 470
   7. Morbid Anatomy of Typhus Fever in Paris, Geneva, &c. By Dr. Lombard .. 471
2. Dr. C. B. Williams on Percussion .. .. .. .. .. .. .. .. .. .. .. .. .. 472
3. Recent Researches on the Sense of Taste.
   1. Experiments of Dr. Alcock on the Glosso-Pharyngeal, the Gustatory, Meckel's Ganglion, and the Naso-Palatine Nerves .. .. .. .. .. .. .. .. .. 474

2. Remarks on Dr. Alcock's Experiments. By D. Noble, Esq. .. .. .. .. .. 474

3. On the Functions of Meckel's Ganglion, and of the Glosso-Pharyngeal Nerve. By M. W. Hilles, Esq. .. .. .. .. .. .. .. .. .. .. .. .. .. .. .. 474

A. Division of the Gustatory Nerves in the Dog .. .. .. .. .. .. .. .. 475

B. Division of the Glosso-Pharyngeal Nerves .. .. .. .. .. .. .. .. .. 475

C. Division of the Branches of the Spheno-Palatine Ganglion .. .. .. .. .. 476

D. Division of the Gustatory and Palatine Nerves .. .. .. .. .. .. .. .. 476

4. Emmenagogue Properties of the Hydriodate of Potass .. .. .. .. .. .. .. 479

5. On Cerebral and Spinal Apoplexy, Paralysis, &c. of New Born Infants. By Dr. Kennedy .. .. .. .. .. .. .. .. .. .. .. .. .. .. .. .. 479

A. Spinal Apoplexy.. .. .. .. .. .. .. .. .. .. .. .. .. .. .. 480

B. Convulsion of the New-Born .. .. .. .. .. .. .. .. .. .. .. .. 480

6. On Contagious Typhus. By Dr. Perry .. .. .. .. .. .. .. .. .. .. 481

7. Ulceration of the Brain .. .. .. .. .. .. .. .. .. .. .. .. .. 482

8. Circumscribed False Aneurism of the Brachial Artery, caused by Puncture in Blood-letting, and cured by Operation. By Mr. Wall.. .. .. .. .. .. .. 483

9. Muriate of Barytes for White Swellings .. .. .. .. .. .. .. .. .. 484

10. Extraordinary Deformity of the Pelvis .. .. .. .. .. .. .. .. .. 485

11. Mr. Rankin on Croton Oil in Spleen .. .. .. .. .. .. .. .. .. .. 486

12. Pemphygus Simplex .. .. .. .. .. .. .. .. .. .. .. .. .. .. 486

13. Retention of Urine after Amputation of the Penis.. .. .. .. .. .. .. 487

14. Change of Form in the Head, concurrently with Change of Character and Talents 488

15. Hunteriana .. .. .. .. .. .. .. .. .. .. .. .. .. .. .. .. .. 489

1. Difficulties of Genius .. .. .. .. .. .. .. .. .. .. .. .. .. 490

2. John Hunter among the Gods .. .. .. .. .. .. .. .. .. .. .. .. 491

3. John Hunter preserved from being made an Old Woman .. .. .. .. .. 491

4. Encouragement given to Science by the English Government .. .. .. 491

5. Hunter an indifferent Lecturer .. .. .. .. .. .. .. .. .. .. .. 492

6. Hunter not a mere Operator .. .. .. .. .. .. .. .. .. .. .. .. 493

7. Hunter's Early Rising and Little Sleep .. .. .. .. .. .. .. .. .. 493

8. His Infirmity of Temper .. .. .. .. .. .. .. .. .. .. .. .. 493

16. Case of obscure Disease .. .. .. .. .. .. .. .. .. .. .. .. .. 495

### Spirit of the Foreign Periodicals, &c.

1. Discovery of the Natural Cow-Pox at Passy near Paris—Report of M. Bousquet.. 497

2. Case of Glanders .. .. .. .. .. .. .. .. .. .. .. .. .. .. .. .. 500

3. Example of Spontaneous Combustion. Remarks on this Extraordinary Phenomenon 500

4. Signor Catonoso's Case of Ligature of the Axillary Artery, below the Clavicle .. 504

5. Dr. Zarlenza on the Cholera at Naples .. .. .. .. .. .. .. .. .. .. 505

6. Abstract of M. Chomel's Clinique at the Hôtel Dieu .. .. .. .. .. .. .. 506

A. Intermittent Fevers .. .. .. .. .. .. .. .. .. .. .. .. .. .. 507

B. Exanthemata .. .. .. .. .. .. .. .. .. .. .. .. .. .. .. .. 507

7. Illustrations of Thoracic Pathology—Connexion of Diseases of the Heart with Acute Rheumatism, &c... .. .. .. .. .. .. .. .. .. .. .. .. .. .. .. 511

1. Fatal Case of Acute Rheumatism—Appearances on Dissection .. .. .. 511

2. M. Cazaneuve on Endocarditis .. .. .. .. .. .. .. .. .. .. .. .. 513

3. Rare Case of Hydro-Pneumato-Pericardium .. .. .. .. .. .. .. .. .. 514

4. Case of Empyema—Paracentesis Thoracis—Recovery .. .. .. .. .. .. 514

5. Compression of the Thorax recommended in the Treatment of Pulmonary Vomicæ.. .. .. .. .. .. .. .. .. .. .. .. .. .. .. .. .. 515

6. On the Pneumonia of Old People. By M.M. Hourmann and Dechambre .. 515

7. Remarks on Infantile Asthma, connected with an Enlarged State of the Thymus Gland, with Cases.. .. .. .. .. .. .. .. .. .. .. .. .. 520

8. On the Nature and Seat of Angina Pectoris .. .. .. .. .. .. .. .. 522

9. On the Cause of the First Act of Respiration in the New-Born Infant.. .. 525

8. Illustrations of Abdominal Pathology.

1. Cases of Abscess of the Liver—in one instance Associated with Hydatids—communicating with the Lungs .. .. .. .. .. .. .. .. .. .. .. .. 527

2. Cases of Abscess in the Abdominal Parietes. By Dr. Gorgone of Palermo.. 528

3. Memorable Case of Spontaneous Perforation of the Bowels .. .. .. .. .. 530

4. On the Enlargements of the Spleen, after Agues and other Fevers .. .. 531

9. Hygiene Publique. Par A. J. Parent Duchatelet [continued from p. 410.] .. .. 537

### Clinical Review, and Hospital Reports.

North London Hospital.

1. How to Pare a Bottle Nose.. .. .. .. .. .. .. .. .. .. .. .. .. .. .. 541

Glasgow Royal Infirmary.

2. Fracture of the Neck of the Thigh Bone, external to the Capsule .. .. .. .. .. 542

Western Lying-in Hospital.

3. On the Length of the Umbilical Cord, and its Mechanical Influence on Parturition, 543

Kent and Canterbury Hospital.

4. Case of Typhoid Fever, accompanied by a diseased state of the Blood, &c. By J. M'Divitt, M.D. .. .. .. .. .. .. .. .. .. .. .. .. .. .. .. .. 545
5. Statistics of the Rich and Poor .. .. .. .. .. .. .. .. .. .. .. .. .. .. 549

Meath Hospital.

6. Clinical Lectures of Dr. Graves .. .. .. .. .. .. .. .. .. .. .. .. .. .. 550

   *a.* Epidemic Erysipelas .. .. .. .. .. .. .. .. .. .. .. .. .. .. .. 550

   *b.* Enemata of Tartar Emetic in the Delirium of Fever .. .. .. . . .. 551

   *c.* Thirst of Fever.. .. .. .. .. .. .. .. .. .. .. .. .. .. .. .. 551

   *d.* Necessity of Anticipating Cerebral Symptoms .. .. .. .. .. .. . 552

Bath Casualty Hospital.

7. Dissection of the Limb, 21 years after the External Iliac Artery was Tied. By G. Norman, Esq... .. .. .. .. .. .. .. .. .. .. .. .. .. .. .. .. .. 552

Guy's Hospital.

8. On the Situation and Structure of Malignant Diseases of the Liver .. .. .. 553
9. Case of Hydatids Discharged from the Lungs.. .. .. .. .. . . .. .. .. .. 555
10. Wound of the Ulnar Artery—Hæmorrhage restrained by Pressure on the Radial and Ulnar .. .. .. .. .. .. .. .. .. .. .. .. .. .. .. .. .. .. 557
11. On the Reputed Cure of Hydrocele by Puncture .. .. .. .. .. .. .. .. 557

Salisbury Infirmary.

12. Case of Recovery from the Insensibility of Intoxication, by the Performance of Tracheotomy. By S. Samson, Esq. .. .. .. .. .. .. .. .. .. .. .. .. .. 560

Marylebone Infirmary.

13. Case of Recovery from the Influence of Opium, by Artificial Respiration. By C. Smith, Esq. . .. .. .. .. .. .. .. .. .. .. .. .. .. . .. .. 562
14. Midwifery in the French Lying-in Hospitals .. .. .. .. .. . .. .. .. 563
15. Clinical Cases of Morbid Growths from the Bones of the Face, &c. .. .. .. 563
    1. Case of Exostosis of the Bones of the Face. By Mr. Morgan .. .. .. 563
    2. Case of a large Bony Tumor of the Face, removed by Spontaneous Separation. By Mr. Hilton .. .. .. .. .. .. .. .. .. .. .. .. .. .. 564
16. Clinical Observations of the late M. Dupuytren on Contusion, &c. of the Heart .. 567
    *a.* Traumatic Compression of the Heart .. .. .. .. .. .. .. .. .. .. 568
    *b.* Contusion of the Heart .. .. .. .. .. .. .. .. .. .. .. .. .. .. 568
    *c.* Displacement of the Heart .. .. .. .. .. .. .. .. .. .. .. .. .. 568
17. Opinions of a French Surgeon on the State of the Ophthalmic Hospitals of London 569

Norfolk and Norwich Hospital.

18. Report of Surgical Patients admitted in 1834. By J. G. Johnson, Esq... .. .. 571

    *a.* Case of Compound Fracture of the Skull .. .. .. .. .. .. .. .. .. 572

    *b.* Fracture of the Basis .. .. .. .. .. .. .. .. .. .. .. .. .. .. 573

    *c.* Compound Fracture and Depression of the Skull.. .. : .. .. .. 574

19. Sketch of the Hospitals of the British Auxiliary Legion in Spain .. .. .. .. 574

### Miscellanies.

1. Medical Attendance on the Sick Poor.
   1. On the Arrangements connected with the Relief of the Sick Poor. By J. Yelloly, M. D. .. .. .. .. .. .. .. .. .. .. .. .. .. .. .. .. 579
   2. On the Present Condition of Medical Relief for the Sick Poor .. .. .. 579
      *a.* Applicability of the Principle of Competition .. .. .. .. .. .. .. 579
2. Poison of Pork. By Dr. M'Divitt .. .. .. .. .. .. .. .. .. .. .. .. .. 584

### Extra-Limites.

1. Irish Medical Profession .. .. .. .. .. .. .. .. .. .. .. .. .. .. .. 585

2. Efficacy of Lobelia Inflata in Inflam. of the Mucous Lining of the Bronchial Tubes. 586

3. Selection from the Laws, &c. of the British Medical Association .. .. .. .. .. 587

4. Case of Iritis and Temporary Blindness from Tic-Douloureux. By D. Jamison, M.D. 589

5. Reclamations.. .. .. .. .. .. . .. .. .. .. .. .. .. .. .. .. .. 590

Bibliographical Record .. .. .. .. .. .. .. .. .. .. .. .. .. .. .. .. 591

